# Athletes with high knee abduction moments show increased vertical center of mass excursions and knee valgus angles across sport-specific fake-and-cut tasks of different complexities

**DOI:** 10.3389/fspor.2022.983889

**Published:** 2022-09-26

**Authors:** Kevin Bill, Patrick Mai, Steffen Willwacher, Tron Krosshaug, Uwe G. Kersting

**Affiliations:** ^1^Institute of Biomechanics and Orthopaedics, German Sport University Cologne, Cologne, Germany; ^2^Department of Mechanical and Process Engineering, Offenburg University, Offenburg, Germany; ^3^Oslo Sports Trauma Research Center, Department of Sports Medicine, Norwegian School of Sports Sciences, Oslo, Norway

**Keywords:** anterior cruciate ligament, biomechanics, change of direction, cutting, injury prevention, knee loading, screening, unanticipated

## Abstract

Young female handball players represent a high-risk population for anterior cruciate ligament (ACL) injuries. While the external knee abduction moment (KAM) is known to be a risk factor, it is unclear how cutting technique affects KAMs in sport-specific cutting maneuvers. Further, the effect of added game specificity (e.g., catching a ball or faking defenders) on KAMs and cutting technique remains unknown. Therefore, this study aimed: (i) to test if athletes grouped into different clusters of peak KAMs produced during three sport-specific fake-and-cut tasks of different complexities differ in cutting technique, and (ii) to test whether technique variables change with task complexity. Fifty-one female handball players (67.0 ± 7.7 kg, 1.70 ± 0.06 m, 19.2 ± 3.4 years) were recruited. Athletes performed at least five successful handball-specific sidestep cuts of three different complexities ranging from simple pre-planned fake-and-cut maneuvers to catching a ball and performing an unanticipated fake-and-cut maneuver with dynamic defenders. A *k*-means cluster algorithm with squared Euclidean distance metric was applied to the KAMs of all three tasks. The optimal cluster number of *k*_*optimal*_ = 2 was calculated using the average silhouette width. Statistical differences in technique variables between the two clusters and the tasks were analyzed using repeated-measures ANOVAs (task complexity) with nested groupings (clusters). KAMs differed by 64.5%, on average, between clusters. When pooling all tasks, athletes with high KAMs showed 3.4° more knee valgus, 16.9% higher downward and 8.4% higher resultant velocity at initial ground contact, and 20.5% higher vertical ground reaction forces at peak KAM. Unlike most other variables, knee valgus angle was not affected by task complexity, likely due to it being part of inherent movement strategies and partly determined by anatomy. Since the high KAM cluster showed higher vertical center of mass excursions and knee valgus angles in all tasks, it is likely that this is part of an automated motor program developed over the players' careers. Based on these results, reducing knee valgus and downward velocity bears the potential to mitigate knee joint loading and therefore ACL injury risk.

## Introduction

In team sports, the majority of ACL injuries are non-contact in nature (1–3), and a subset of these are formed by cutting maneuvers (2). Young female handball players are at greater risk compared to their male counterparts (1, 4, 5). From a biomechanical perspective, the external knee abduction moment (KAM) has been identified as a risk factor and is likely to be a contributing factor in the injury mechanism (2, 6–11). Increases in KAMs have the potential to elevate knee valgus which, in turn, results in a shift in the axial force toward the lateral compartment of the knee. These lateral compressive forces might provoke internal rotation of the tibia (12). Markolf et al. (13) have been able to show *in vitro* that this combination of knee valgus and tibia internal rotation substantially increases ACL strain.

While the KAM can be calculated to give insight into the risk for future injuries, it is unclear how handball players with different KAMs differ in terms of their cutting technique. Several technique parameters have been identified as possible predictors for KAMs during simple change-of-direction tasks (14–16), but these test scenarios are not game-specific and might therefore not be suitable as tasks to understand the causes for high KAMs. In team handball, fake-and-cut maneuvers aiming to fake a cut in a certain direction to subsequently pass an opponent in the other direction have been identified as a common injury mechanism (17).

Kristianslund et al. (18) used a linear regression to predict knee abduction moments. Technique variables, such as cut width at initial contact (IC), cut angle, knee valgus angle at IC, foot strike angle at IC, and the approach speed, explained most of the variance in the KAM magnitudes. A linear regression approach works well when the relationships are truly linear, however, these relationships may very well be different for athletes with high compared to low KAMs. Thus, clustering athletes based on their KAM magnitudes is likely a more robust approach for understanding what distinguishes athletes with high KAMs from the remaining athletes.

Reduced decision times during a choice reaction task (19) and the presence of a simulated defensive opponent (8) have been shown to influence KAMs and knee valgus angles, respectively. However, it is unclear how cutting technique variables are affected by the task complexity, i.e., how reduced anticipation times or the addition of key game elements such as ball handling or opponent interaction affect cutting technique in more sport-specific cutting maneuvers. Identifying differences in the cutting technique over multiple tasks of varying complexity could provide valuable insight into the design of and the screening protocols for in-field screenings and identify motor programs that might put athletes at higher risk for sustaining an ACL injury.

Therefore, the purpose of the study was to group athletes into clusters based on their peak KAM magnitudes produced in three fake-and-cut tasks of varying complexity, and to test for differences in cutting technique variables between the identified clusters. Furthermore, we investigated whether the task complexity affects the technique variables. Understanding how technique variables affect knee joint loading in sport-specific tasks might be beneficial in the development of ACL injury prevention programs.

## Materials and methods

### Participants

Fifty-one female handball players (mean ± SD: 67.0 ± 7.7 kg, 1.70 ± 0.06 m, 19.2 ± 3.4 years) from various Norwegian handball clubs (elite division, 1st, 2nd, or 3rd division; Appendix 1) were recruited. The included players typically train 4–5 times per week (3rd division players) and up to 10–11 times per week (elite players attending elite sports schools). All athletes were at least 16 years of age and played the back, line, or wing position (Appendix 2). All athletes were injury- and pain-free at the time of testing. The University Ethics Committee approved the study prior to data collection, and written consent was obtained from all players.

### Experimental setup and protocol

Eighty-two retro-reflective markers of a full-body marker set were attached to each athlete. The lower extremity markers were attached to the following anatomical landmarks: left and right anterior superior iliac spines and posterior superior iliac spines; medial and lateral femoral condyles; medial and lateral malleoli. Tracking clusters attached to a rigid shell consisting of four markers were attached to the lateral aspect of the thigh and the shank of both legs. Rearfoot markers were placed on the athletes' shoes at the most medial, lateral, and posterior aspects of the calcaneus. Forefoot markers were attached to the shoe upper at the head of the first and fifth metatarsal and the distal hallux. Upper body markers were attached to the vertebra prominens (C7), the 10th vertebra of the thoracic spine, jugular notch of the clavicle, and xiphoid process of the sternum (trunk segment). Additionally, tracking markers were placed on the forearms, upper arms, hands, and head (20). Following marker attachment, a standardized warm-up procedure was followed, including 5 min of cycling, various side shufflings, ten jump squats, seven squats, and seven calf raises.

A three-dimensional (3D) marker-based tracking system (24 cameras, Qualisys, Gothenburg, Sweden, 200 Hz) and two floor-embedded force plates (AMTI, Watertown, Massachusetts, USA, 600 x 1200 mm, 1000 Hz) sampled the marker trajectories and the ground reaction forces (GRFs) of the athletes during three standardized cutting tasks of different complexities. For all tasks, the players accelerated for 6 m and arrived at an angle of approximately 35° to the long axis of the runway. Athletes performed all tasks at self-selected speeds while they were instructed to match a representative game intensity. Prior to data collection, athletes were allowed to familiarize themselves with each cutting task.

**Figure 1:**
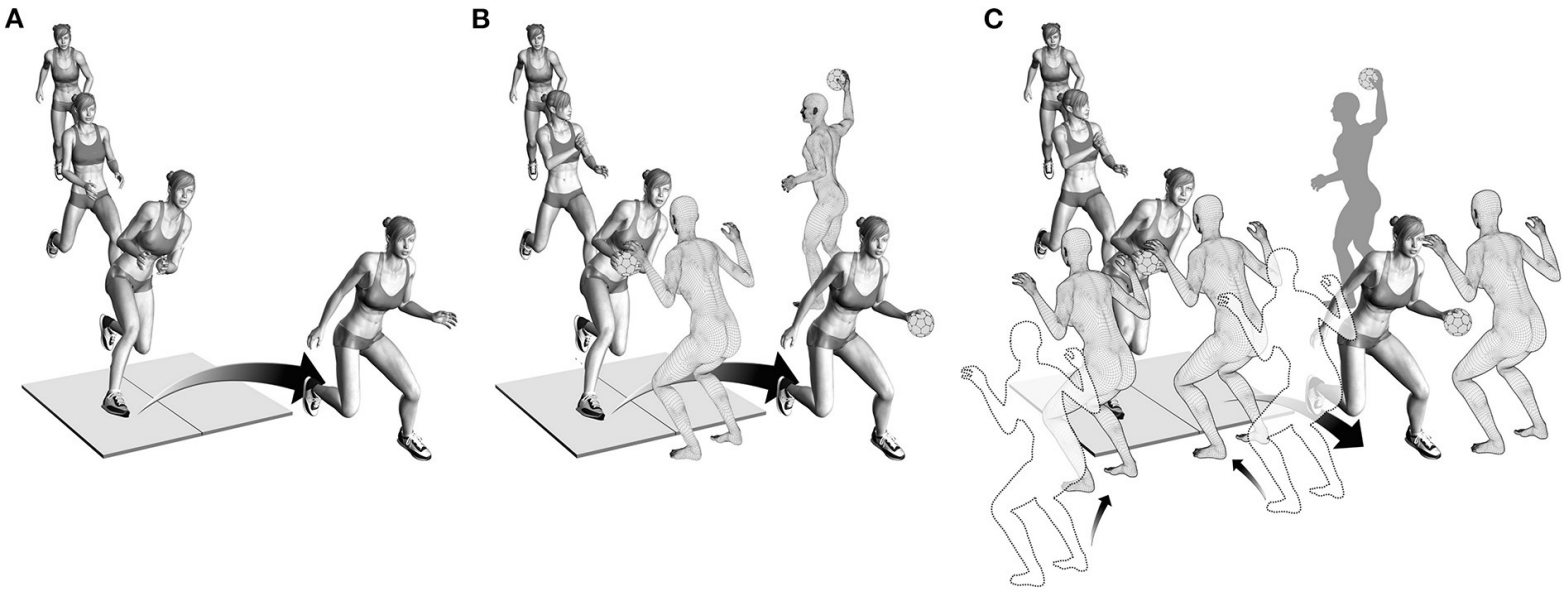
**(A)** Illustration of Task 1. Players approached the force plate and performed a pre-planned fake-and-cut maneuver. **(B)** Illustration of Task 2. Players caught a ball passed by a teammate while approaching the force plate and subsequently performed a pre-planned fake-and-cut maneuver in front of a static defender. **(C)** Illustration of Task 3. Players caught the ball passed by a teammate while the middle and one randomly alternating outside defender moved toward the athlete to block one side. This scenario forced the athlete to cut to the unblocked side, resulting in an unanticipated cut.

For Task 1, the athletes were instructed to perform a pre-planned fake-and-cut maneuver, similar to what they would do during active gameplay (Figure 1A). There was no ball or defender involved. Task 2 was performed the same way as Task 1, but with the added elements of catching a ball passed by an experienced handball player one step before initiating a pre-planned fake and cut in front of a static defender (Figure 1B) (18). For Task 3, a defender was added to either side of the static defender of Task 2. The middle defender and one randomly alternating outside defender moved toward the athlete at the instance of the catch, forcing the athlete to cut away from the dynamic defenders. This scenario resulted in an unanticipated cut (Figure 1C).

The order of the three tasks was randomized. A minimum of five valid cuts per task was recorded. A cut was considered valid if the foot landed clearly within the boundaries of one force plate. For Tasks 2 and 3, the defenders' positions were tracked using a retro-reflective marker attached to their backs. Cutting leg for Tasks 1 and 2 was determined based on playing position and throwing arm, resulting in *n* = 46 and *n* = 5 athletes performing these tasks on their right and left leg, respectively. Since the cutting direction for Task 3 was unanticipated, the fake-and-cut task was performed on both the left and right leg. However, only the leg determined for Tasks 1 and 2 was analyzed.

### Data analysis

A recursive 4th order low-pass Butterworth filter with 20 Hz cut-off frequency was applied to the raw marker trajectories and ground reaction forces (21, 22). Knee and ankle joint centers were defined as the midpoints between medial and lateral femoral condyles and malleoli markers, respectively. Hip joint centers and coordinate systems were defined according to Bell et al. and Seidel et al. (23, 24). Segment inertial properties were calculated based on anthropometric data derived from de Leva (25). Lower extremity resultant external joint moments were determined with the explicit expression provided by Hof (26) using a rigid body model of the lower extremities. All model calculations were performed using a custom-made MATLAB script (R2021a, The Mathworks, Natick, USA). Details of these calculations can be found in previous publications (20, 27).

Peak external KAM within the first 100 ms after IC was normalized to body mass. The time window between 0 and 100 ms after IC was selected as it represents a time window in which most non-contact ACL injuries occur (2, 6, 28). IC and toe-off (TO) were defined as the time points at which the unfiltered vertical GRF component exceeded or fell below 30 N, respectively. Initiation of the block by the defenders of Task 3 was defined as the instance when the individual defender's marker velocity reached 0.5 m/s. The athlete's time to decide on a cutting direction and plan the cutting maneuver was calculated as the time difference between the initiation of the block by the outside defenders and the athlete's IC.

### Technique variables

**Figure 2:**
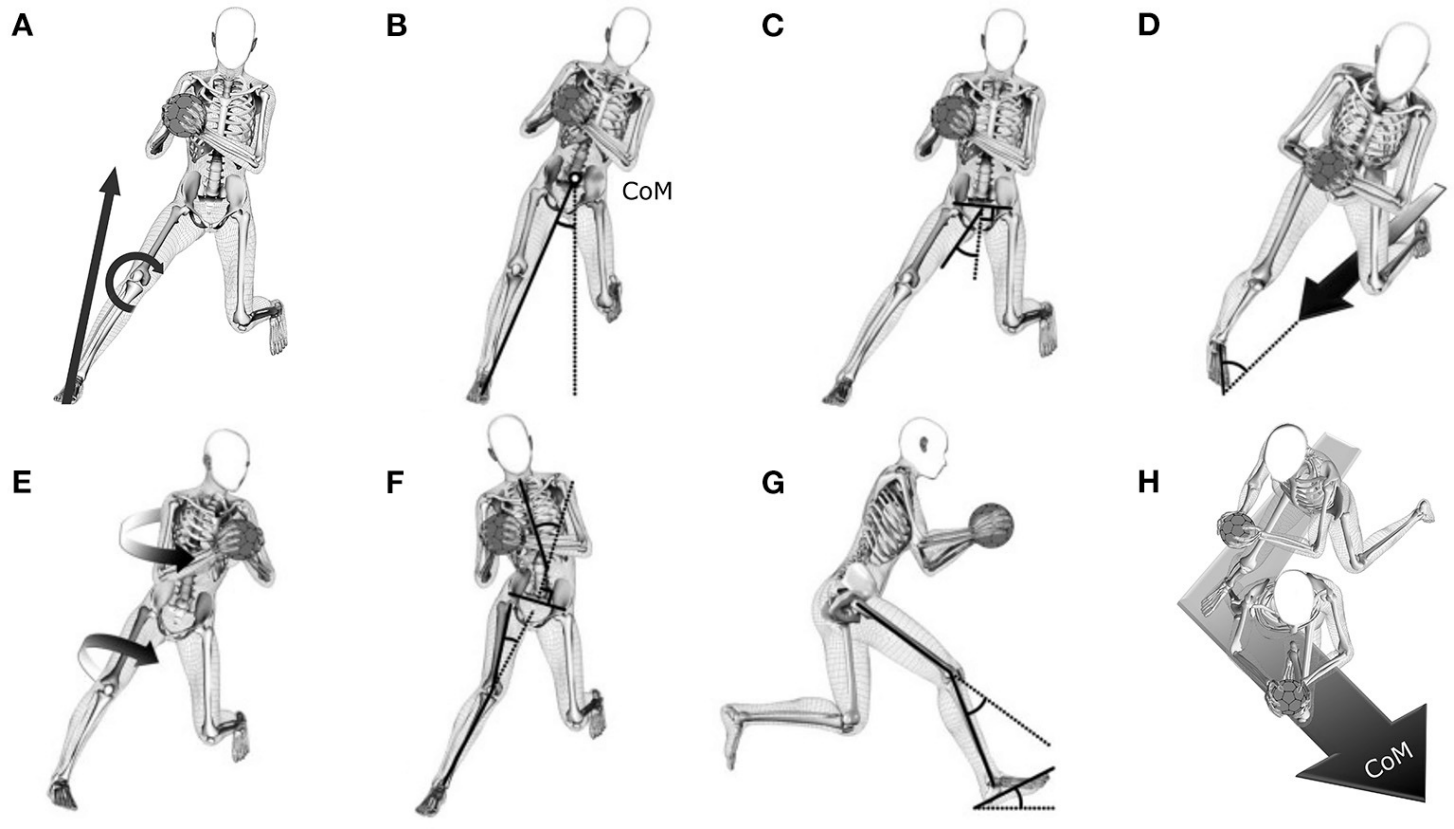
**(A)** Illustration of the external knee abduction moment (KAM) and technique variables described at initial contact; **(B)** cut width; **(C)** hip abduction; **(D)** foot progression angle; **(E)** trunk rotation and hip internal rotation; **(F)** trunk lateral flexion and knee valgus; **(G)** knee flexion and foot strike angle; **(H)** cut angle. Additional technique factors were trunk rotation angular velocity, horizontal center of mass (CoM) velocity, resultant CoM velocity and its anteriorly-, laterally-, and vertically-directed components, and contact time.

The cutting technique was described according to a previous publication by Kristianslund et al. (18). The following kinematic variables at IC were determined: foot strike angle, foot progression angle, knee flexion angle, knee valgus angle, hip abduction angle, hip rotation angle, trunk lateral flexion angle, and trunk rotation angle as well as trunk rotation angular velocity (Figure 2). Horizontal (center of mass; CoM) velocity and resultant (CoM) velocity and its anterior, lateral, and vertical components were derived from the 3D CoM trajectories. Other technique variables included the vertical GRF at peak KAM, cut angle, cut width, and ground contact time.

The foot strike angle was defined as the angle between the long axis of the foot and its projection onto the ground (18) (Figure 2G), with positive values indicating forefoot landing. Foot progression angle was defined as the angle between the long axis of the foot projected onto the horizontal plane and the vector of the horizontal CoM velocity, with negative values indicating an externally rotated foot relative to the horizontal velocity vector (Figure 2D). For knee valgus angle, higher positive values indicate an increase in valgus (Figure 2F). For hip abduction angle, higher positive values indicate more abduction (Figure 2C). For hip rotation angle, negative values indicate external rotation (Figure 2E). Trunk lateral flexion angle (Figure 2F) and trunk rotation angle (Figure 2E) were calculated as angles between the pelvis segment relative to the trunk segment. Positive values for trunk lateral flexion angles indicate lateral flexion of the trunk relative to the pelvis toward the side of the cutting leg, whereas rotation of the trunk relative to the pelvis toward the cutting leg is indicated by negative trunk rotation angles. Horizontal velocity was defined as the vector sum of the absolute CoM velocities in anterior and lateral directions. Resultant velocity was defined as the vector sum of the absolute 3D CoM velocities. Negative values for the vertical velocity indicate downward movement. Cut angle (Figure 2H) was defined as the angle between the horizontal velocity vector at IC and TO. Cut width (Figure 2B) was defined as the angle between a line from the point of force application (PoFA) to the center of mass and the vertical in a plane perpendicular to the direction of movement 5 ms after IC (18).

### Statistics

A *k*-means cluster analysis with squared Euclidean distance metric was used to identify athletes with similar KAM amplitudes. The input for the analysis was the *n*-by-*p* data matrix containing the average peak KAM in Nm/kg within the first 100 ms of stance for each subject (*n* = 51) in each task (*p* = 3), resulting in a 3D feature space with 51 data points (Appendix 3). Clustering was performed for *k* ranging from 2 to 20, with cluster center initialization following a *k*-means++ algorithm (29). Cluster centroids were recalculated until convergence was achieved. Clustering for each *k* was performed 200 times, and the solution with the lowest total sum of distances among all the replicates for *k* was used. The optimal value for *k* (*k*_*optimal*_) was determined based on the highest average silhouette width of the clustered data (30). The silhouette method was chosen since overfitting is less likely because unreasonably increasing the cluster number will lead to data points getting closer to other clusters which, in turn, will reduce the average silhouette width.

After grouping the athletes into *k*_*optimal*_clusters based on the KAM amplitudes, repeated-measures ANOVAs (task complexity) with nested grouping (cluster) were applied to identify the effects of the clusters (cluster effect) and the task complexity (task effect) on cutting technique variables. Clusters-by-task-complexity effects (interaction effects) served as an indicator if the influence of the cluster on a cutting technique variable was dependent on the task.

Since all analyzed technique variables are discrete variables, those with significant cluster effects were further analyzed to gain additional insight into these variables. Repeated-measures ANOVAs with nested grouping of statistical parametric mapping (SPM, v.M0.4.8, www.spm1d.org) (31) were applied to test for significant effects of cluster and task as well as their interaction effect on these technique variables between IC and 100 ms of stance.

All data were tested for normality. *Post-hoc* tests for discrete parameters were performed with Bonferroni correction, and the level of significance for all statistical tests was set to α = 0.05.

## Results

### Cluster analysis

**Figure 3:**
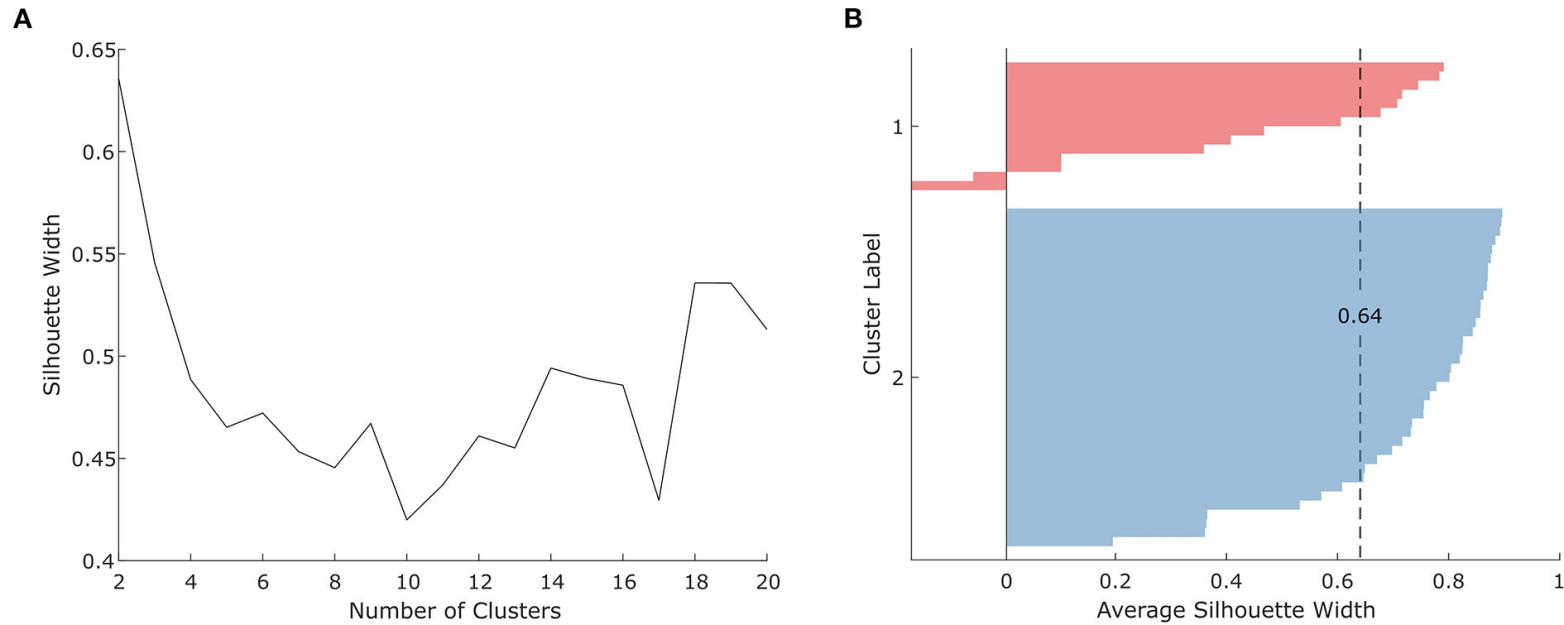
**(A)** Silhouette scores for 2–20 clusters and **(B)** silhouette analysis for the optimal number of clusters (*k*_*optimal*_= 2).

The optimal number of clusters based on the athletes' peak KAMs within the first 100 ms of stance in the three tasks was found to be *k*_*optimal*_ = 2 with an average silhouette width of 0.64 (Figure 3A). Clusters 1 and 2 included *n* = 14 and *n* = 37 athletes, respectively (Figure 3B). Athletes assigned to either of the two clusters were similar in age, body height, and body mass, as shown by the results of independent *t*-tests (Table 1). In Cluster 1, one athlete had previously sustained an ACL injury to the non-cutting leg but no athlete (0% of Cluster 1) reported a previous ACL injury to the cutting leg. Cluster 2 contained six athletes with a previous ACL injury, two (5.4%) of which had either sustained the injury to the cutting leg (*n* = 1) or both legs (*n* = 1). For details on previous ACL injuries, see Appendix 4.

**Table 1:** Descriptive demographics and anthropometrics for athletes of both clusters.

	**Cluster 1**	**Cluster 2**	***t*** **-Test**
**Parameter**	**Mean ±SD**	**Mean ±SD**	***p*** **-value**
Age [years]	21.0 ± 3.7	18.3 ± 1.8	0.06
Body Height [cm]	169.8 ± 6.9	172.7 ± 5.4	0.70
Body Mass [kg]	68.5 ± 7.2	69.2 ± 8.0	0.45

### Peak knee abduction moments within the first 100 ms of stance

**Figure 4:**
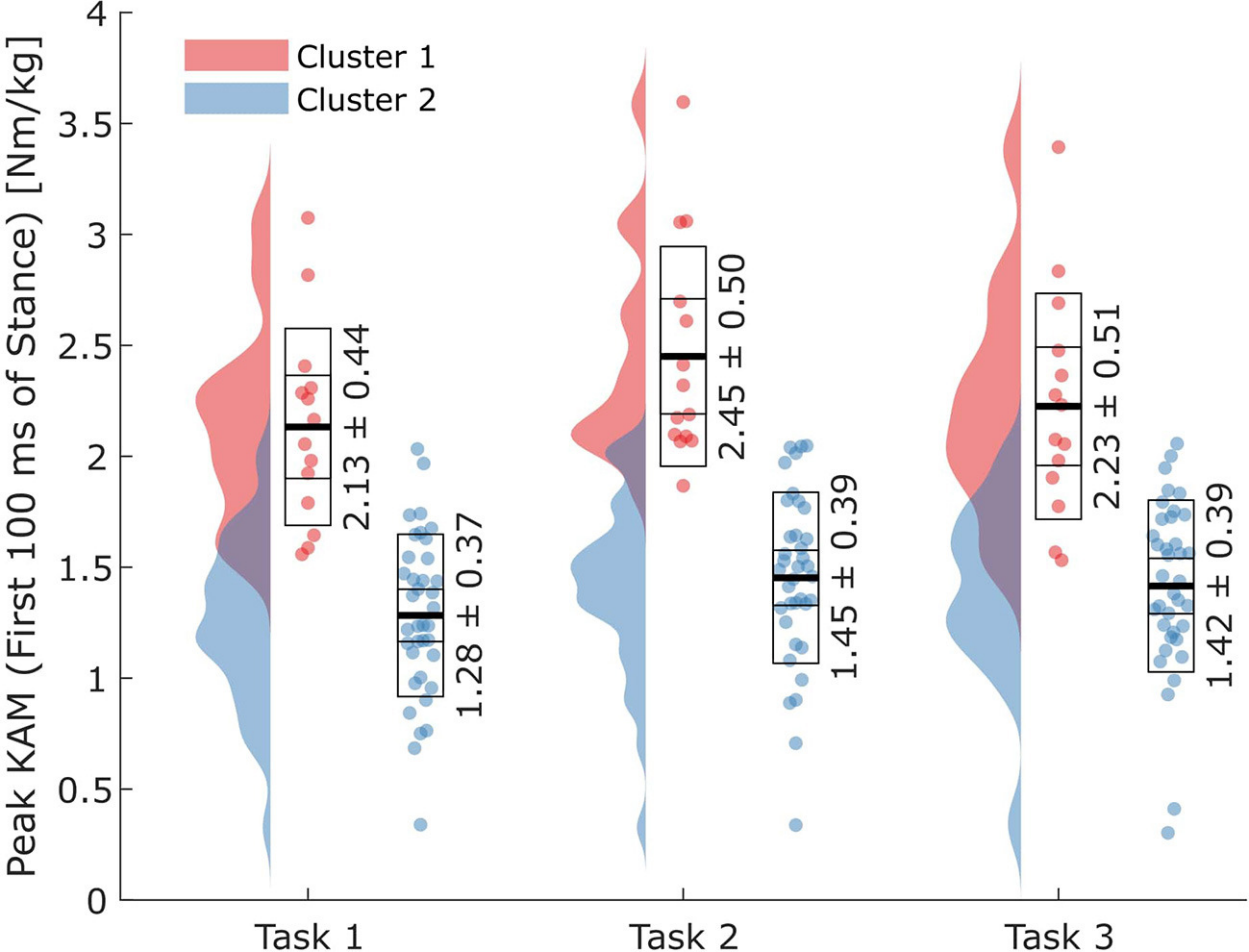
Peak external knee abduction moments (KAMs) within the first 100 ms after initial ground contact for the two clusters and three task complexities. Horizontal lines in the boxes on the data point clouds represent (from inside to outside) the means (thick lines), standard errors of the mean, and standard deviations.

Athletes assigned to Cluster 1 showed significantly higher peak KAMs than athletes assigned to Cluster 2. On average, athletes assigned to Cluster 1 produced 0.89 Nm/kg (64.5%) higher peak KAMs compared to athletes assigned to Cluster 2 when pooling all three tasks (Cluster 1: 2.27 ± 0.49 Nm/kg, Cluster 2: 1.38 ± 0.38 Nm/kg; *p*_*cluster*_ < 0.001; Figure 4; Table 2). Further, a significant task effect (*p*_*task*_ < 0.001) was observed. For both clusters, Task 2 resulted in the highest peak KAMs (Cluster 1: 2.45 ± 0.50 Nm/kg, Cluster 2: 1.45 ± 0.39 Nm/kg), and Task 1 resulted in the lowest peak KAMs (Cluster 1: 2.13 ± 0.44 Nm/kg, Cluster 2: 1.28 ± 0.37 Nm/kg). No interaction effect was identified (*p*_*interaction*_ = 0.257).

**Table 2:** Means and standard deviations for the analyzed technique variables in the three tasks separated by the two identified clusters, and results for the nested repeated-measures ANOVAs with cluster main effect, task main effect, and cluster-by-task interaction effect.

**Variable**	**Task 1**		**Task 2**		**Task 3**		**Cluster**	**Task**	**Interaction**
							**effect**	**effect**	**effect**
	**Cluster 1**	**Cluster 2**	**Cluster 1**	**Cluster 2**	**Cluster 1**	**Cluster 2**	***p_*cluster*_***	***p_*task*_***	***p_*interaction*_***
Peak KAM (First 100 ms of stance) [Nm/kg]	2.13 ± 0.44	1.28 ± 0.37	2.45 ± 0.50	1.45 ± 0.39	2.23 ± 0.51	1.42 ± 0.39	**< 0.001**	**< 0.001**	0.26
Foot strike angle at IC [°]	3.5 ± 14.2	3.1 ± 12.0	−3.5 ± 14.6	−1.6 ± 13.2	8.5 ± 9.4	6.1 ± 10.1	0.93	**< 0.001**	0.35
Foot progression angle at IC [°]	−9.1 ± 3.0	−7.8 ± 4.6	−8.4 ± 6.3	−9.9 ± 4.7	−11.3 ± 4.0	−10.4 ± 4.8	0.96	**0.002**	0.18
Knee flexion angle at IC [°]	24.8 ± 6.1	24.5 ± 6.5	22.7 ± 4.3	23.0 ± 5.5	24.3 ± 5.5	24.4 ± 8.4	1	**0.016**	0.92
Knee valgus angle [°] at IC	8.4 ± 3.2	5.5 ± 3.7	8.4 ± 3.2	4.8 ± 3.0	9.0 ± 3.6	5.5 ± 3.1	**< 0.001**	0.11	0.60
Hip abduction angle at IC [°]	18.1 ± 3.3	17.1 ± 4.6	18.2 ± 3.4	17.8 ± 5.5	18.8 ± 4.0	18.8 ± 5.0	0.73	**0.006**	0.56
Hip rotation angle at IC [°]	−0.7 ± 6.2	−2.3 ± 4.8	−1.8 ± 5.4	−1.7 ± 5.0	−0.3 ± 4.0	−1.9 ± 4.8	0.55	0.72	0.15
Trunk lateral flexion angle at IC [°]	7.4 ± 7.2	8.4 ± 5.9	6.2 ± 6.8	6.4 ± 6.7	7.1 ± 6.2	8.1 ± 6.5	0.70	**0.028**	0.85
Trunk rotation angle at IC [°]	−16.1 ± 11.2	−19.1 ± 10.1	−26.1 ± 12.3	−20.7 ± 11.3	−20.4 ± 9.2	−20.7 ± 11.1	0.82	**0.014**	**0.024**
Trunk rotation velocity at IC [°/s]	−87.7 ± 91.0	−64.2 ± 94.2	−90.1 ± 106.1	−111.6 ± 114.5	−88.6 ± 97.6	−84.6 ± 100.5	0.95	**0.008**	0.19
Horizontal CoM velocity at IC [m/s]	2.97 ± 0.41	2.89 ± 0.33	3.37 ± 0.42	3.08 ± 0.28	3.07 ± 0.43	2.92 ± 0.31	0.09	**< 0.001**	0.10
Resultant CoM velocity at IC [m/s]	3.49 ± 0.26	3.28 ± 0.29	3.75 ± 0.32	3.43 ± 0.26	3.55 ± 0.33	3.26 ± 0.26	**< 0.001**	**< 0.001**	0.33
Anterior CoM velocity [m/s]	2.75 ± 0.42	2.68 ± 0.33	3.13 ± 0.39	2.87 ± 0.30	2.87 ± 0.41	2.75 ± 0.31	0.11	**< 0.001**	0.06
Lateral CoM velocity [m/s]	1.10 ± 0.23	1.03 ± 0.32	1.11 ± 0.31	1.07 ± 0.32	1.06 ± 0.30	0.95 ± 0.27	0.35	**0.046**	0.80
Vertical CoM velocity [m/s]	1.79 ± 0.32	1.52 ± 0.32	1.67 ± 0.30	1.49 ± 0.26	1.74 ± 0.28	1.42 ± 0.30	**0.004**	**0.025**	0.12
Vertical GRF at peak KAM [N/kg]	31.26 ± 5.81	26.20 ± 5.13	32.61 ± 4.95	26.95 ± 5.24	30.92 ± 5.97	25.52 ± 6.17	**0.001**	**0.038**	0.90
Cut angle [°]	70.7 ± 13.1	70.9 ± 14.6	65.2 ± 13.4	70.8 ± 15.2	58.7 ± 15.6	62.6 ± 13.4	0.44	**0.002**	0.29
Cut width at IC [°]	21.2 ± 3.1	21.0 ± 2.3	21.8 ± 2.9	21.3 ± 2.8	22.6 ± 3.5	22.9 ± 2.9	0.89	**< 0.001**	0.71
Contact time [s]	0.31 ± 0.05	0.30 ± 0.05	0.30 ± 0.06	0.31 ± 0.04	0.29 ± 0.05	0.28 ± 0.04	0.95	**0.002**	0.22

Bold values indicate significant effects.

### Technique variables

**Figure 5:**
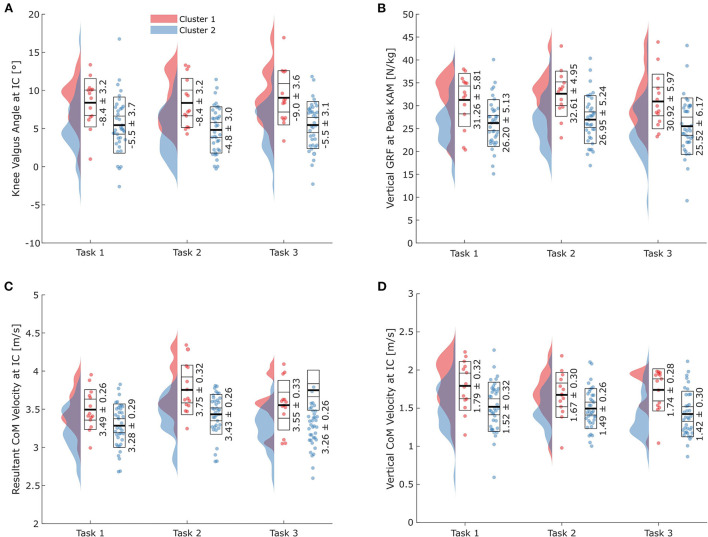
Results for all three tasks for the technique variables with significant cluster effects. **(A)** Knee valgus angle at initial ground contact (IC); **(B)** vertical ground reaction force (GRF) at peak knee abduction moment (KAM); **(C)** resultant center of mass (CoM) velocity at IC; **(D)** vertical CoM velocity at IC. Horizontal lines in the boxes on the data point clouds represent (from inside to outside) the mean (thick line), standard error of the mean, and standard deviation.

Significant cluster effects were found for the knee valgus angle at IC (*p*_*cluster*_ = 0.001), the vertical GRF at peak KAM (*p*_*cluster*_ < 0.001), the resultant velocity at IC (*p*_*cluster*_ < 0.001), and the vertical velocity at IC (*p*_*cluster*_ = 0.004) (Figures 5A–D). When pooling tasks, athletes in Cluster 1 displayed greater knee valgus angles at IC (+3.4°), higher vertical GRFs at peak KAM (+5.38 N/kg), higher resultant velocities at IC (+0.28 m/s), and higher vertical velocities at IC (-0.25 m/s corresponding to an increased downwards velocity) than athletes in Cluster 2 (Figures 5A–D; Table 2).

Of the 18 variables analyzed, 16 showed significant (*p*_*task*_ < 0.05) task effects, with knee valgus angle at IC and hip rotation angle at IC being the only exceptions (Table 2). Results for the pairwise comparisons between the tasks can be found in Appendix 4.

The trunk rotation angle at IC was the only variable with a significant cluster-by-task interaction effect (*p*_*interaction*_ = 0.024). While differences between the clusters were marginal in Task 3, athletes in Cluster 1 displayed 3° more trunk rotation toward the cutting leg in Task 1. In contrast, athletes in Cluster 1 produced 5.4° more trunk rotation in Task 2.

**Figure 6:**
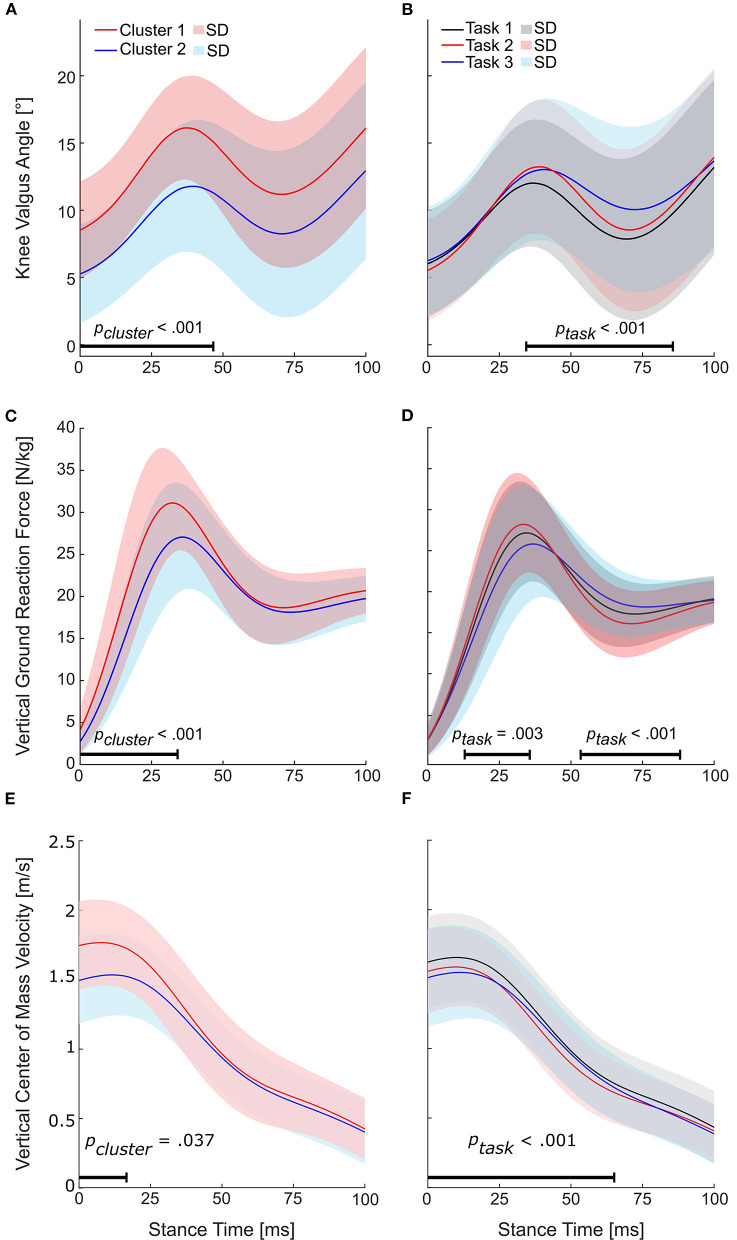
Results for the nested repeated-measures ANOVAs of statistical parametric mapping (SPM). Horizontal bars indicate the range with a significant cluster (*p*_*cluster*_ < 0.05) or task effect (*p*_*task*_ < 0.05). **(A)** Time series for the knee valgus angles of the identified clusters and **(B)** tasks. **(C)** Time series for the vertical ground reaction forces of the identified clusters and **(D)** tasks. **(E)** Time series for the vertical center of mass velocities of the identified clusters and **(F)** tasks.

Nested repeated-measures ANOVAs of SPM performed for the knee valgus angle, vertical GRF, and vertical velocity revealed significant cluster (*p*_*cluster*_ < 0.05) and task effects (*p*_*task*_ < 0.05) for all three variables (Figures 6A–F). For the trunk rotation angle, the nested repeated-measures ANOVA of SPM revealed no significant cluster effect (p_cluster_ >0.05) but a significant task effect (*p*_*task*_ < 0.05).

### Attacker-defender distance and reaction time

**Figure 7:**
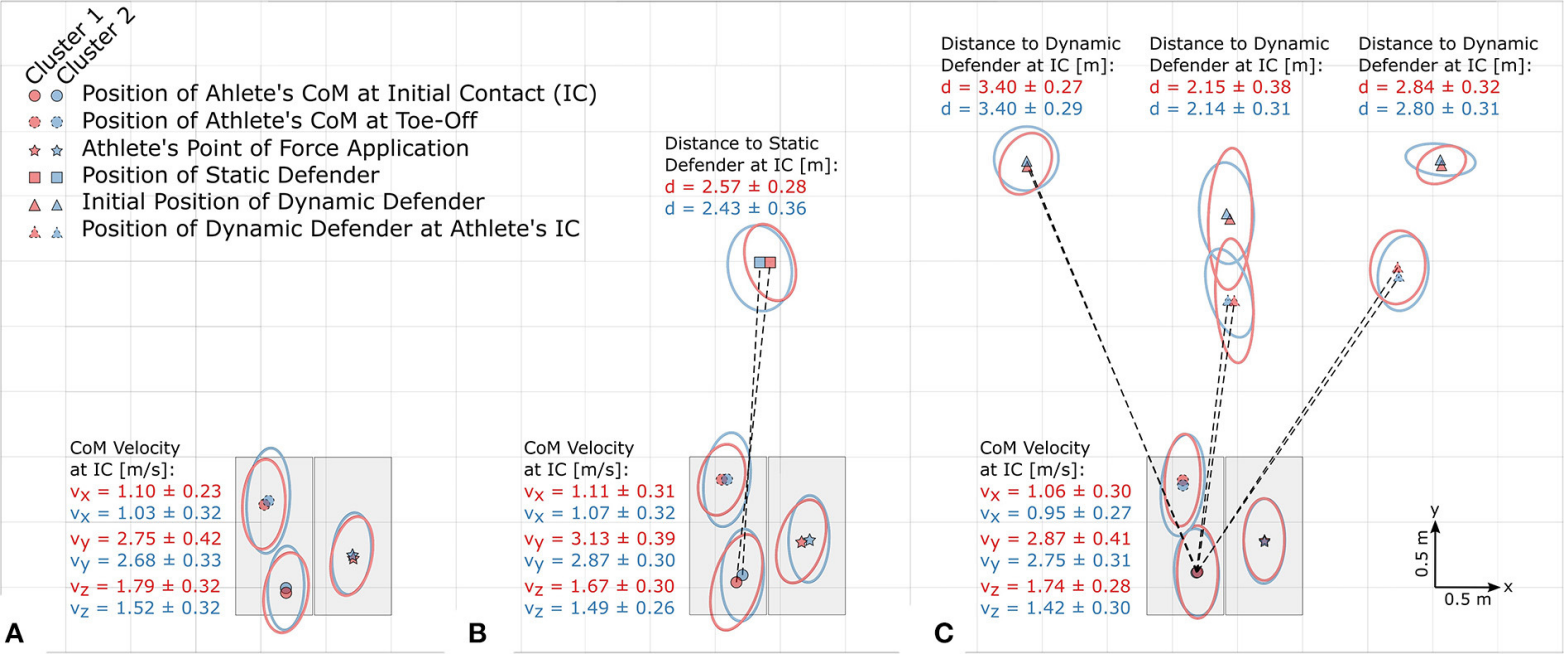
Bird's eye view (true to scale) of **(A)** Task 1, **(B)** Task 2, and **(C)** Task 3. Data of left-foot cuts (*n* = 75) were transformed into the coordinate systems of athletes performing right-foot cuts. Ellipses around the symbols represent the standard deviations.

An analysis of the distance to the static defender at IC in Task 2 revealed a 5.5% shorter distance (2.43 ± 0.36 m vs. 2.57 ± 0.28 m, *p* < 0.001) for athletes of Cluster 2 compared to Cluster 1. In Task 3, no statistically significant differences in the distance to the middle defender (Cluster 1: 2.15 ± 0.38 m, Cluster 2: 2.14 ± 0.31 m, *p* = 0.817) or time to react (Cluster 1: 0.92 ± 0.16 s, Cluster 2: 0.94 ± 0.14 s, *p* = 0.234) were found (Figure 7).

## Discussion

The main aim of this study was to identify differences in cutting technique variables between athletes grouped into clusters based on their peak KAMs within the first 100 ms of stance produced during three sport-specific fake-and-cut tasks of different complexities. The optimal number of clusters *(k*_*optimal*_*)* was found to be 2, with an average silhouette width of 0.64. The mean relative differences in KAMs between the clusters when pooling all three tasks was 64.5%. Cluster 1 athletes showed 20.5% higher vertical GRFs at peak KAM, 3.4° higher knee valgus angles at IC, as well as 8.4% higher resultant and 16.9% higher vertical CoM velocity at IC. These findings imply that athletes can reduce their KAMs by avoiding valgus positions and high vertical impact velocities. As we assume that increased knee valgus and vertical CoM velocities are part of an athlete's inherent movement strategy which is likely developed over many years of playing handball, focusing on reducing these potential risk factors should start at an early stage of the playing career.

The higher valgus angles in Cluster 1 compared to Cluster 2 were consistently larger and lasted throughout the first 47 ms of stance, and higher vertical GRFs lasted throughout the first 35 ms of stance. The vertical CoM velocity, on the other hand, was only higher within the first 20 ms for athletes with high KAMs, indicating that the kinetic energy was dissipated in a short time period, leading to high impact forces. Hence, these variables are likely to contribute to ACL loading in a period in which ACL injury typically occurs (2, 6, 28). Thus, importantly, these findings suggest that the discrete values at IC are an indicator of increased peak KAM and ACL injury risk.

The higher downward velocity for athletes in Cluster 1 means that these athletes' vertical CoM excursions prior to the IC must have been higher. This is confirmed by visual inspection of the recorded data, which shows a pronounced “jump” onto the force plate for athletes with higher vertical and resultant velocities. An analysis of the time to reach the peak KAM shows, on average, 9.3% shorter times for athletes in Cluster 1 (37 ± 11 ms) compared to Cluster 2 (40 ± 12 ms) when pooling the three tasks. It is plausible that the identified jump of the athletes showing higher KAMs is a strategy aimed at gaining time to “read” where the defender might be moving and/or to decide on the next move without breaking the three-step-rule (a player can only do three steps before having to throw the ball). Therefore, exercises aiming at reducing reaction/decision times might help reduce KAMs without compromising the speed at which the cut is executed. While preliminary deceleration has been suggested as a strategy to reduce horizontal deceleration and knee joint loading experienced during subsequent steps (32), the same strategy might be beneficial for athletes using a jump as a tool to gain time to decide on a cutting direction. Focusing on a cutting technique that minimizes the downward CoM velocity at IC seems preferable in order to reduce the generated impulse.

Importantly, recent developments in in-field markerless motion analysis might make the factors associated with high KAMs (i.e., knee valgus angle and vertical velocity) possible to be measured during a real game scenario as no force data are required. Thus, our findings may be used to assess risk in-game or during training. The higher vertical velocities at IC for athletes with higher KAMs indicate the necessity for higher decelerating forces, possibly explaining the differences in the vertical GRFs and making the challenging on-field collection of forces redundant.

We believe that incorporating landing stabilization exercises, as recommended in a systematic review with meta-analysis by Petushek et al. (33), as well as strength training into an athlete's training program can be possible ways to reduce knee valgus and therefore KAMs. Although strength assessments were not part of the present study, previous studies have shown that increasing hip muscle strength can reduce ACL injury risk (34).

Of the 18 technique variables analyzed, 16 were shown to be significantly affected by the task complexity. Interestingly, knee valgus angle at IC was one of the variables not affected by the task complexity. Hence, it is possible that knee valgus is either part of a player's anatomy or inherent movement strategy that has been internalized over their careers.

Since the majority of the more KAM-promoting technique variable magnitudes are found in Task 2, this task might be best suited to resolve differences in cutting techniques between clusters. The resultant velocity at IC and the vertical GRF at peak KAM, two of the three variables that were both significantly affected by the cluster and the task complexity, showed their highest magnitudes in that task. Adding the other eight variables with the highest magnitudes in Task 2, although not significantly affected by the cluster, has on aggregate likely led to Task 2 producing the highest KAMs. It needs to be noted that for some variables (e.g., foot strike angle where positive values correspond to forefoot landing) higher values might decrease KAMs (18). Foot strike angle was therefore counted as one of the ten variables with the highest (=KAM-promoting) magnitudes in Task 2 since subjects showed, on average, a rearfoot landing pattern in that task (unlike in Tasks 1 and 3), which has been linked to increases in KAMs (18). As low knee flexion angles have been shown to contribute to increased ACL loading (13), knee flexion angle at IC was counted toward the variables with the highest (= KAM-promoting) magnitudes in Task 2.

Although our results show that technique variables depend on task complexity, differences in magnitudes between tasks were generally well within intra-task standard deviations and might therefore not be clinically relevant. The only exception is the foot strike angle which shows a forefoot landing pattern in Tasks 1 and 3, whereas most athletes landed on their rearfoot in Task 2. Since Task 2 accounts for only one-third of the data, but cluster effects were calculated using the pooled data of all tasks, potential cluster effects on the foot strike angle in Task 2 might have been diluted by the additional data of Tasks 1 and 3.

Several different approaches to investigate associations between cutting techniques and KAMs have been presented in the literature. Similar to our findings, Kristianslund et al. reported that knee valgus angles had the highest impact on KAM, and knee valgus at IC was identified as a high-risk posture for ACL injury in a systematic review (35). These findings give confidence that reducing the knee valgus is crucial in reducing the KAM and likely the most important factor associated with high KAMs although Krosshaug et al. (36) showed that this variable cannot be used for screening purposes in vertical drop jumps. The resultant velocity was also found to be a factor that highly contributed to the KAM in the study by Kristianslund et al., and the same was found in the present study, adding to the body of evidence for the absolute velocity to be a driving force in increasing the KAM. Our breakdown into the three velocity components shows that the downward velocity of the CoM was the main factor in separating the two clusters in regard to their CoM velocities. However, in contrast to the findings of Kristianslund et al., the two clusters identified in the present study did not differ significantly in terms of their cut width, foot strike angle, or cut angle. For these three variables, the standard deviation within a cluster and task was a multiple of the differences in the mean magnitude of the respective variable between the clusters, indicating that the differences between clusters were much smaller than the one standard deviation increases in the study by Kristianslund et al. (18). This also means that isolated changes to a single variable may alter the KAM; however, isolated changes to technique variables are most likely not common, and an interdependence between technique variables is likely. In other words, a change in a specific variable is probably accompanied by other changes that might either counteract or magnify the effect of the change in a variable on the KAM. While linear regression models have the potential to uncover the relationships between input (cutting technique) and output (KAM) variables in linearly separable datasets, the possible presence of multicollinearity of variables is a disadvantage of linear regression models which might explain why high and low KAM athletes did not differ in terms of their cut width, foot strike angle or cut angle in the present study. Further, the present study included two additional tasks, and data were pooled using all three tasks. In contrast, the study by Kristianslund et al. (18) included only one task (Task 2).

While clustering based on the technique variables is also possible, the number of subjects in the present study is likely insufficient for such an approach. Furthermore, we expect more than two clusters to be present when using 18 technique variables as input for the analysis, and therefore expect the interpretability of the data to be more complex. Another approach could have been to compare players above/below a certain KAM threshold, based on, e.g., risk factor or cadaver studies. However, such approaches might not be straightforward, since we hypothesized that the KAM would be task-dependent, and therefore thresholds must be individually adjusted to each task.

While this study focused on biomechanical variables differing between clusters of different KAM magnitudes, clustering based on other risk factors than the KAM might identify other technique variables as different between identified clusters. We want to highlight the fact that classifying athletes into clusters based on their KAM magnitudes, as done in the present study, serves the aim of identifying biomechanical technique and motor pattern differences between KAM clusters rather than identifying a threshold above which athletes are at an increased risk for injuries.

A few limitations need to be considered. A total of seven athletes had a previous ACL injury. However, only two of them sustained the injury to the analyzed leg. Both of these athletes were assigned to Cluster 2 and accounted for 5.4% of athletes in that cluster. We visually compared these athletes' knee kinematics time curves to the uninjured athletes, which did not reveal any abnormalities. We further compared their minimum and maximum knee joint angles in all three planes to the mean minimum and maximum values of the cohort. All six values for either of the two athletes fell within one standard deviation of the cohort. We therefore conclude that including these athletes in the study did not affect the interpretation of our results.

While the average silhouette width of 0.64 was fairly high, seven of the 14 subjects assigned to Cluster 1 showed values lower than that, two of which showed negative silhouette widths. The negative silhouette widths of the two subjects indicate that they were likely misclassified (30). Therefore, a secondary analysis was performed in which these athletes were moved to the other cluster. Doing this washed away the task effect observed for the lateral velocity at IC (*p*_*task*_ = 0.060 instead of *p*_*task*_ = 0.046) and added an interaction effect for the anterior velocity at IC (*p*_*interaction*_ = 0.027 instead of *p*_*interaction*_ = 0.063), but cluster effects were not affected. Although the number of athletes tested for this study is relatively small, the results of the secondary analysis suggest the approach selected is robust against falsely detecting cluster effects.

## Conclusion

The vast majority of technique variables change with task complexity; however, these changes are generally well below intra-task standard deviations and might therefore be negligible. The exception to this is the foot strike angle, which seems to be strongly affected by task complexity. Athletes with high KAMs show 3.4° more knee valgus, 20.5% higher vertical GRFs, 8.4% higher resultant CoM velocities, and 16.9% higher vertical CoM velocities during handball-specific cutting maneuvers. Aiming at reducing the downward CoM velocity and reducing the knee valgus angle should be a main focus in ACL injury prevention programs in team handball.

## Data availability statement

The original contributions presented in the study are included in the article/Supplementary material, further inquiries can be directed to the corresponding author/s.

## Ethics statement

The studies involving human participants were reviewed and approved by Regional Ethics Committee of the Norwegian School of Sports Sciences. Written informed consent to participate in this study was provided by the participants' legal guardian/next of kin.

## Author contributions

KB and PM contributed to data acquisition, data processing, data analysis, and the writing of the manuscript. SW contributed to the writing of the manuscript. TK contributed to project planning, design of the study, and the writing of the manuscript. UK assisted in the design of the study, the data analysis, and contributed to the writing of the manuscript. All authors contributed to the article and approved the submitted version.

## Funding

The Oslo Sports Trauma Research Center has been established at the Norwegian School of Sport Sciences through generous grants from the Royal Norwegian Ministry of Culture, the South-Eastern Norway Regional Health Authority, the International Olympic Committee, the Norwegian Olympic Committee & Confederation of Sport, and Norsk Tipping AS.

## Conflict of interest

The authors declare that the research was conducted in the absence of any commercial or financial relationships that could be construed as a potential conflict of interest.

## Publisher's note

All claims expressed in this article are solely those of the authors and do not necessarily represent those of their affiliated organizations, or those of the publisher, the editors and the reviewers. Any product that may be evaluated in this article, or claim that may be made by its manufacturer, is not guaranteed or endorsed by the publisher.
