# Supplementary material for: Athletes with high knee abduction moments show increased vertical center of mass excursions and knee valgus angles across sport-specific fake-and-cut tasks of different complexities
Source: Front Sports Act Living. 2022 Sep 26;4:983889. doi: 10.3389/fspor.2022.983889 (PMC9548628; doi:10.3389/fspor.2022.983889)
Supplement: Supplementary file 1 [file Data_Sheet_1.PDF]

Athletes With High Knee Abduction Moments Show Increased Vertical Center of Mass Excursions And Knee Valgus Angles Across Sport-Specific Fake-And-Cut Tasks of Different Complexities

Kevin Bill[1*], Patrick Mai[1,2], Steffen Willwacher[1,2], Tron Krosshaug[3], Uwe G. Kersting[1]

[1]Institute of Biomechanics and Orthopaedics, German Sport University Cologne; Germany
[2]Department of Mechanical and Process Engineering, Offenburg University; Germany
[3]Oslo Sports Trauma Research Center, Department of Sports Medicine, Norwegian School of Sports Sciences

*** Correspondence:**
k.bill@dshs-koeln.de

Appendix 1. League affiliation of athletes.

	Cluster 1		Cluster 2		Total	
League	*n*	Relative Share [%]	*n*	Relative Share [%]	*n*	Relative Share [%]
REMA 1000-ligaen*	2	14.3	4	10.8	6	11.8
First Division	2	14.3	5	13.5	7	13.7
Second Division	6	42.9	15	40.5	21	41.2
Third Division	2	14.3	5	13.5	7	13.7
Junior	1	7.1	7	18.9	8	15.7
Other	1	7.1	1	2.7	2	3.9

Appendix 2. Field positions of athletes. Numbers do not add up to 14 (Cluster 1), 37 (Cluster 2) and 51 (total) due to athletes playing multiple positions.

	Cluster 1		Cluster 2		Total	
Position	*n*	Relative Share [%]	*n*	Relative Share [%]	*n*	Relative Share [%]
Back	10	71.4	31	83.8	41	80.4
Line	2	14.3	2	3.9	4	7.8
Wing	3	21.4	11	29.7	14	27.4

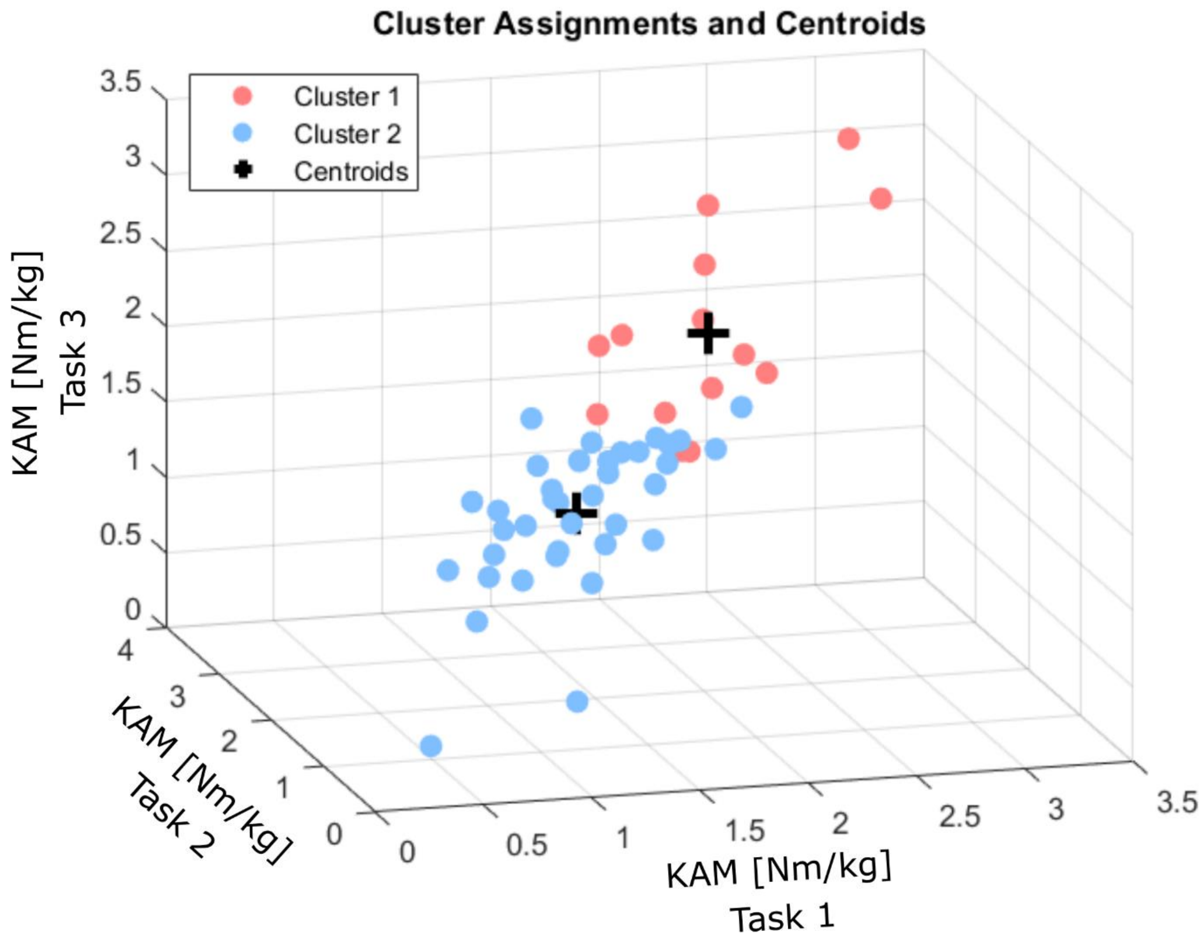

Appendix 3. Cluster assignments and centroids based on the average peak external knee abduction moments within the first 100 ms of stance. Each data point represents one subject.

Appendix 4. Number of athletes with previous ACL injuries.

Previous ACL Injury	Cluster 1 *n*	Cluster 1 Relative Share [%]	Cluster 2 *n*	Cluster 2 Relative Share [%]	Total *n*	Total Relative Share [%]
Cutting Leg	0	0	1	2.7	1	2.0
Non-Cutting Leg	1	7.1	4	10.8	5	9.8
Both Legs	0	0	1	2.7	1	2.0

Appendix 5. Technique variables and their magnitudes for all three tasks, results of repeated-measures ANOVAs and pairwise comparisons.

				Task Effect	Pairwise Comparison		
Variable	Task 1	Task 2	Task 3	p_{task}	Task 1 vs. 2	Task 1 vs. 3	Task 2 vs. 3
Peak KAM (First 100 ms of Stance) [Nm/kg]	1.52 ± 0.54	1.73 ± 0.61	1.64 ± 0.56	**< .001**	**.003**	**.022**	.37
Foot Strike Angle at IC [°]	3.2 ± 12.5	-2.1 ± 13.5	6.8 ± 9.9	**< .001**	**< .001**	**.010**	**< .001**
Foot Progression Angle at IC [°]	-8.2 ± 4.2	-9.5 ± 5.2	-10.7 ± 4.6	**.002**	.27	**< .001**	.16
Knee Flexion Angle at IC [°]	24.6 ± 6.3	22.9 ± 5.1	24.3 ± 7.7	**.016**	**.023**	1	.06
Knee Valgus Angle [°] at IC	6.3 ± 3.8	5.8 ± 3.4	6.5 ± 3.6	.11	–	–	–
Hip Abduction Angle at IC [°]	17.4 ± 4.2	17.9 ± 4.9	18.8 ± 4.7	**.006**	0.41	**.017**	.166
Hip Rotation Angle at IC [°]	-1.9 ± 5.2	-1.7 ± 5.0	-1.6 ± 4.6	.72	–	–	–
Trunk Lateral Flexion Angle at IC [°]	8.1 ± 6.2	6.4 ± 6.7	7.8 ± 6.4	**.028**	.06	1	.06
Trunk Rotation Angle at IC [°]	-18.2 ± 10.4	-22.2 ± 11.7	-20.6 ± 10.5	**.014**	**.019**	.33	.69
Trunk Rotation Velocity at IC [°/s]	-70.7 ± 93.0	-105.7 ± 111.7	-85.7 ± 98.8	**.008**	**.034**	.46	.10
Horizontal CoM Velocity at IC [m/s]	2.91 ± 0.35	3.15 ± 0.34	2.96 ± 0.35	**< .001**	**< .001**	.64	**< .001**
Resultant CoM Velocity at IC [m/s]	3.34 ± 0.29	3.52 ± 0.31	3.34 ± 0.31	**< .001**	**< .001**	1	**< .001**
Anterior CoM Velocity [m/s]	2.70 ± 0.35	2.94 ± 0.35	2.78 ± 0.34	**< .001**	**< .001**	.19	**< .001**
Lateral CoM Velocity [m/s]	1.05 ± 0.30	1.08 ± 0.32	0.98 ± 0.28	**.046**	1	.46	.07
Vertical CoM Velocity [m/s]	1.59 ± 0.34	1.54 ± 0.28	1.51 ± 0.32	**.025**	.32	**.046**	.71
Vertical GRF at Peak KAM [N/kg]	27.59 ± 5.73	28.51 ± 5.71	27.00 ± 6.53	**.038**	.41	.87	**.034**
Cut Angle [°]	70.8 ± 14.0	69.2 ± 14.9	61.5 ± 14.0	**.002**	.78	**< .001**	**< .001**
Cut Width at IC [°]	21.1 ± 2.5	21.5 ± 2.8	22.8 ± 3.1	**< .001**	.75	**< .001**	**.008**
Contact Time [s]	0.30 ± 0.05	0.31 ± 0.05	0.29 ± 0.04	**.002**	.32	**.008**	**< .001**
